# The SMC-like RecN protein is at the crossroads of several genotoxic stress responses in *Escherichia coli*

**DOI:** 10.3389/fmicb.2023.1146496

**Published:** 2023-04-24

**Authors:** Adrien Camus, Elena Espinosa, Pénélope Zapater Baras, Parul Singh, Nicole Quenech’Du, Elise Vickridge, Mauro Modesti, François Xavier Barre, Olivier Espéli

**Affiliations:** ^1^CIRB, Collège de France, INSERM U1050, CNRS UMR 7241, Université PSL, Paris, France; ^2^Institute for Integrative Biology of the Cell (I2BC), Université Paris-Saclay, CEA, CNRS, Gif-sur-Yvette, France; ^3^Goodman Cancer Research Centre, McGill University, Montreal, QC, Canada; ^4^Cancer Research Center of Marseille, Department of Genome Integrity, CNRS UMR 7258, INSERM U1068, Institut Paoli-Calmettes, Aix Marseille University, Marseille, France

**Keywords:** RecN, genotoxic, mitomycin C (Mit-C), Bleomycin (BLM), homologous recombination (HR), Tn-seq, sister chromatid cohesion, *uvrA*

## Abstract

**Introduction:**

DNA damage repair (DDR) is an essential process for living organisms and contributes to genome maintenance and evolution. DDR involves different pathways including Homologous recombination (HR), Nucleotide Excision Repair (NER) and Base excision repair (BER) for example. The activity of each pathway is revealed with particular drug inducing lesions, but the repair of most DNA lesions depends on concomitant or subsequent action of the multiple pathways.

**Methods:**

In the present study, we used two genotoxic antibiotics, mitomycin C (MMC) and Bleomycin (BLM), to decipher the interplays between these different pathways in *E. coli*. We combined genomic methods (TIS and Hi-SC2) and imaging assays with genetic dissections.

**Results:**

We demonstrate that only a small set of DDR proteins are common to the repair of the lesions induced by these two drugs. Among them, RecN, an SMC-like protein, plays an important role by controlling sister chromatids dynamics and genome morphology at different steps of the repair processes. We further demonstrate that RecN influence on sister chromatids dynamics is not equivalent during the processing of the lesions induced by the two drugs. We observed that RecN activity and stability requires a pre-processing of the MMC-induced lesions by the NER but not for BLM-induced lesions.

**Discussion:**

Those results show that RecN plays a major role in rescuing toxic intermediates generated by the BER pathway in addition to its well-known importance to the repair of double strand breaks by HR.

## Introduction

Molecular processes that maintain genomic integrity are essential for all organisms. This is necessary because DNA damage from internal or external sources can arise during every round of genome duplication. Pioneer works and recent textbooks tend to attribute a particular DNA damage response (DDR) to each type of lesions (Molecular Biology of the Cell). Omics data, however, showed that genomic alterations and their processing involve broader cellular responses such as cell cycle adaptations, metabolic changes or oxidative stress response for example (Derks et al., 2014). The Genomic Stress Response provides a window of opportunity for DNA repair before cells enter into deadly pathways, including adaptation of the cellular metabolism (Mendoza-Chamizo et al., 2018), cell cycle control, lesion repair and lesion tolerance (Hanawalt, 2015). The various repair pathways sometimes compete with each other to process the same lesion. In this multistep repair scheme, each step creates an intermediate that may constitute a new lesion able to recruit another type of repair machinery. In bacteria, DDR involves error free mechanisms: Photoreactivation, Base Excision Repair (BER), Nucleotide Excision Repair (NER) and Homologous Recombination (HR). DDR also involves error prone mechanisms: Non-homologous End Joining (NHEJ) and Translesion Synthesis (TLS). During replication, DNA alterations (i.e., alkylation, oxidation, Cyclobutane Pyrimidine Dimers, adduct, Interstrand Cross Links, frozen enzymes, repair intermediates or arrested replication forks) may lead to either Single Strand (SS) gaps or Double Strand (DS) breaks. These strand breaks are the most deleterious types of DNA damage. In *E. coli*, they induce the SOS response (Radman, 1975) that will allow the massive production of proteins involved in HR, NER and TLS in every strains and eventually NHEJ in strains that encode it (Bhattacharyya et al., 2018).

To preserve their niche, bacteria frequently produce and secrete antibiotics with genotoxic properties. These antibiotics have been used for clinical application to treat infections or cancers (Matic, 2018). In the present work, we analyzed the Genomic Stress Response induced by two genotoxic antibiotics: Bleomycin and Mitomycin C. Bleomycin (BLM) is a glycopeptide antibiotic and anti-tumor agent isolated from *Streptomyces verticillus* that targets primarily the furanose rings of DNA. In the presence of required cofactors (Fe^2+^ and O_2_), BLM directly causes both single-stranded and double-stranded DNA damage. DNA degradation by BLM is initiated by generating a free radical, in the deoxyribose. At low oxygen tension, oxidized abasic (AP) sites are favored while at high oxygen tension SS and DS breaks predominate (Burger et al., 1981). Abasic sites and SS or DS breaks occur at a 3:1 ratio (Steighner and Povirk, 1990; Chen et al., 2008). DS breaks are suspected to be the major cause of cell death. MMC is a prodrug that requires conversion into hydroquinone to exert DNA toxicity. Hydroquinone interacts with DNA, forming cross-linked DNA-mitomycin adducts. Pioneer works showed that the impact of MMC is dose dependent. At low concentration (less than 0.1 μg/ml), the action of the antibiotic is bacteriostatic, resulting in cell elongation and nucleoid compaction (Lossius et al., 1983), but no apparent effect on cellular macromolecules synthesis. At a high level, MMC is highly bactericidal and almost completely inhibits deoxyribonucleic acid synthesis (Suzuki and Kilgore, 1967).

Earlier studies revealed that HR is essential for survival to both BLM (Kosa et al., 2004) and MMC toxicity (Kushner, 1974; Cupido and Bridges, 1985; Keller et al., 2001). The RecN protein, whose production is induced by the SOS regulon, was found to play important roles for the processing and repair of MMC (Picksley et al., 1984) and BLM (Kosa et al., 2004; Xu et al., 2012) DNA lesions. However, the function of RecN in these two repair processes is not yet understood. RecN is a Structural Maintenance of Chromosome (SMC)-like protein (Pellegrino et al., 2012) that binds on single stand DNA where it can catch a second DNA molecule (Keyamura and Hishida, 2019). *In vitro*, RecN stimulates the ligation of DNA molecules (Reyes et al., 2010). *In vivo*, RecN halts sister chromatid segregation and promotes nucleoid compaction (Odsbu and Skarstad, 2014; Vickridge et al., 2017). Overexpression of RecN is toxic for the cell (Warr et al., 2019) and its level is regulated by the ClpXP proteasome (Nagashima et al., 2006; Neher et al., 2006; Warr et al., 2019). Because RecN interacts with RecA (Vickridge et al., 2017) and both are equivalently required to survive I-SCE 1 mediated DSB, they are generally associated in the same epistatic group (Meddows et al., 2005). Recent data, however, suggests that RecA and RecN may also function in genetically distinct pathways important for DNA repair (Klimova and Sandler, 2020).

In the present study, we took advantage of the involvement of RecN in the repair of two different lesions to investigate its role in the Genomic Stress Response. We demonstrate that RecN differently modifies nucleoid management and sister chromatid dynamics according to the drug considered. In the presence of MMC-induced lesions, RecN requires a pre-processing of the lesions by the NER and its activity on sister chromatids is manifested early in the repair process. By contrast, in the presence of BLM-induced lesions RecN activity does not require NER processing and is manifested later during the recovery phase. TIS analysis revealed that RecN is one of the rare DDR genes involved in the Genomic Stress Response (GSR) of both drugs. We found that the absence of RecN increased the pressure on the BER pathway while concomitantly reducing the importance of homologous recombination. TIS analysis also highlighted the importance of drug tolerance pathways such as efflux systems, oxidative stress management and cell cycle controllers for successful recovery from DNA alterations and how RecN activity moves the equilibrium between different solutions. More generally, this work illustrates that GSR is an integrated processes that cells deploy to create the conditions for their survival.

## Results

### Sister chromatid dynamics in normal and pathological conditions

**FIGURE 1:**
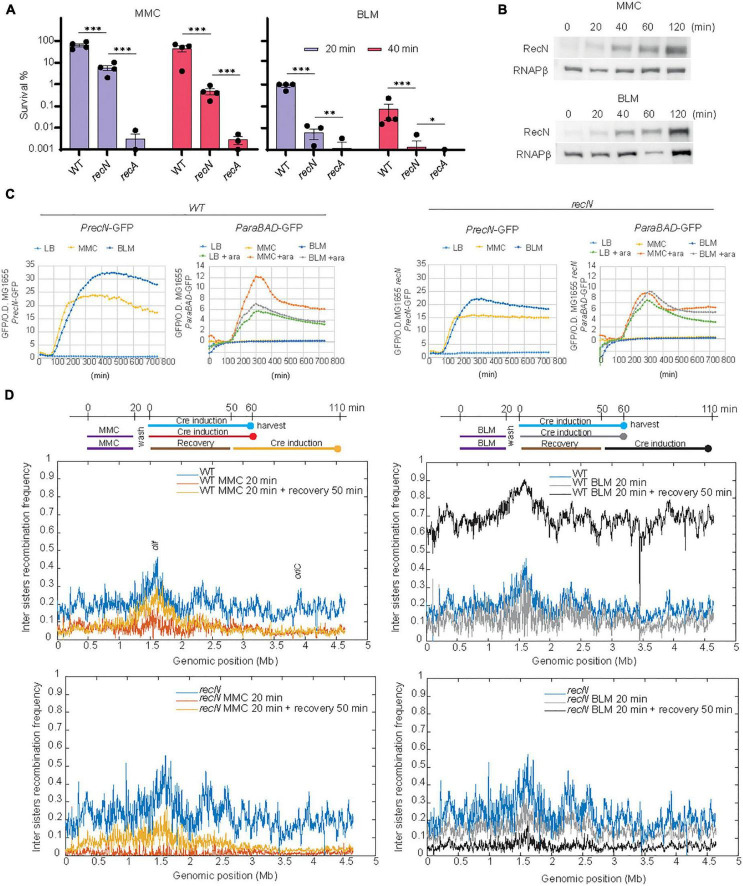
RecN’s influence of sister chromatid interactions differs according to the DNA lesion. **(A)** CFU of WT and *recN* mutant in the presence of MMC (5 μg/mL) or BLM (1 μg/mL). Results are expressed as percentage of survival compared to the untreated condition. **(B)** Induction of RecN in the presence of MMC (5 μg/mL) or BLM (1 μg/mL) measured by western blot. **(C)** SOS and arabinose promoters expression in the presence of MMC and BLM. Both drugs were added at the time point 90 min. **(C)** Hi-SC2 profile of sister chromatid interactions in the WT and *recN* mutant in the presence or absence of MMC or BLM. **(D)** Hi-SC2 inter sisters recombination frequency measures of WT and *recN* strains immediately after a 20 min of MMC or BLM treatment and recovering of their injuries for 50 min before measure. Anova statistical test *<0.05, **<0.005, ***<0.0005, ****<0.00005.

In bacteria, chromosome segregation follows replication, leading to a complete segregation of the origins before the end of the replication round. However, in *E. coli*, imaging of individual genomic loci revealed a short period of cohesion of the two newly replicated sister loci before segregation (Sunako et al., 2001; Espeli et al., 2008; Joshi et al., 2013). Using a genetic tool developed in the lab, we observed sister loci cohesion involves sister chromatid interactions (SCI) that favor recombination (Lesterlin et al., 2012). Using this tool we observed that RecN maintains a high level of SCI in the presence of MMC (Vickridge et al., 2017). Recently, an adaptation of this tool, Hi-SC2, was developed to survey sister chromatid interactions at the genome level (Espinosa et al., 2020a,b). We have seized this opportunity to question SC dynamics at the whole genome level in the presence of MMC and BLM. To find adequate conditions for Hi-SC2, we performed viability assays in the presence of the drugs (Figure 1A). We observed that treatments for 20 min at a concentration of 5 μg/mL of MMC or 1 μg/mL of BLM were tolerated by the WT cells, not tolerated by the *recA* mutant and affected the survival of the *recN* mutant. We controlled that these treatments induced RecN production in the WT and *recN* strains (Figures 1B, C) and did not affect expression of the P*araBAD* promoter that is required to induce Cre recombinase expression in the Hi-SC2 experiment. Variations of inter sister chromatid LoxP/Cre recombination frequency according to genome coordinates were plotted using a 10 kb sliding window (Figure 1D). In the absence of drugs we observed a monotonous recombination frequency (RF) profile with domes (≈ 500 kb) of higher recombination frequency (RF ≈ 20%) separated by valleys (RF ≈ 10%). Overall, the recombination frequency was slightly higher in the terminus domain and near *oriC*. These observations agree with previous analysis in *E. coli* of single loci (Lesterlin et al., 2012). The WT and *recN* profiles were very similar (Pearson Coefficient _*WT/*_*_*recN*_* = 0.83). In the WT strain, we observed that MMC treatment provoked a general decrease of RF (mean RF ≈ 6%) and presumably of SCI. In the absence of RecN this reduction was dramatic and almost no recombination was observed in the conditions of the assay (RF ≈ 1%; PC _*WT–MMC*20/recN–MMC20_ = 0.15, Figure 1D). In the presence of BLM, RF was maintained to a high level in the WT strain (RF ≈ 12%; PC_WT/wt–BLM20_ = 0.77) and decreased modestly in the *recN* mutant (RF ≈ 10%; PC _*recN*/*recN*–BLM20_ = 0.77, Figure 1D). The influence of RecN on SCI may therefore differ according to the DNA damage. The small effect of the *recN* deletion on SCI in the presence of BLM was surprising. For a deeper investigation of this phenomenon, we performed Hi-SC2 experiments during the recovery phase when cells were washed from MMC or BLM (Figure 1D). 50 min after MMC wash, the level of SCI was partially recovered in the WT cells (RF ≈ 8%; PC _*WT/WT–MMC*50_ = 0.69) and the *recN* mutant (RF ≈ 11%; PC _*recN/recN–MMC*50_ = 0.58). In the presence of BLM, we observed a very high recombination frequency in the WT strain (RF ≈ 60%) but correlated with that of the untreated cells (PC _*WT/WT–BLM*50_ = 0.79). In the *recN* strain, by contrast, RF became very low (RF ≈ 5%) during the recovery period. Hi-SC2 analysis confirmed that RecN is required to maintain SCI in the immediate moments following MMC treatment; however, during the recovery phase of MMC-induced damage, RecN does not seem important to restore SCI. We also reveal that RecN does not play a major role during the initial BLM-induced damages’ phase but instead is required during the recovery phase of BLM-induced damages. These observations suggest that RecN intervenes at different time points of the repair strategies for MMC and BLM tolerance.

### Alteration of nucleoid morphology in response to RecN activity

**FIGURE 2:**
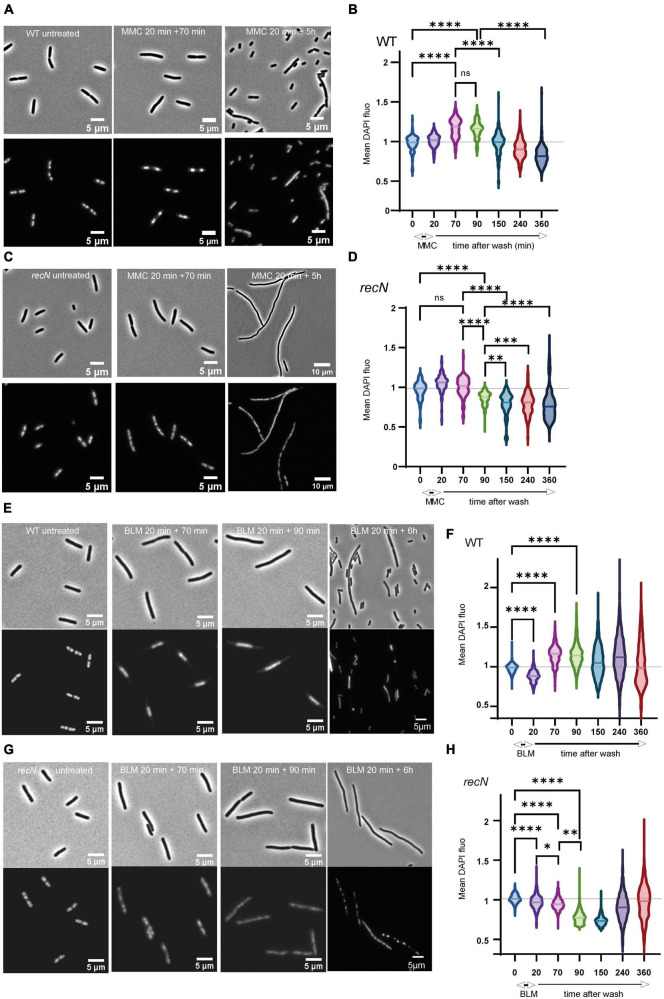
RecN’s influence of nucleoid compaction. **(A)** Observation of cells and nucleoid of WT cells treated with MMC **(B)** quantification of the mean DAPI fluorescence in the nucleoid area of WT cells treated with MMC. Integrated fluorescence amount in the nucleoid divided by the area of the nucleoid is a proxy for nucleoid compaction. **(C)** Same as A for the *recN* mutant. **(D)** Same as B for the *recN* mutant. **(E)** Observation of cells and nucleoid of WT cells treated with BLM. **(F)** Quantification of the mean DAPI fluorescence in the nucleoid area of WT cells treated with BLM. **(G)** Same as D for the *recN* mutant. **(H)** Same as F for the *recN* mutant. *N* = 500, Anova statistical test *<0.05, ^**^<0.005, ^***^<0.0005, ^*⁣*⁣**^<0.00005.

In response to various stresses the bacterial nucleoid compacts transiently in a RecN dependent manner (Keyamura et al., 2013; Odsbu and Skarstad, 2014; Vickridge et al., 2017). Using fluorescence imaging, we questioned whether SCI correlate with nucleoid compaction. We observed cell morphology and nucleoid shape in a time course experiment corresponding to a 20 min treatment followed by 6 h of recovery (Figure 2A). After MMC treatment, WT cells stopped dividing quickly (Supplementary Figure 1) and compacted their nucleoid (time points 70 and 90 min); they recovered nearly completely in 3 h and then entered in an apparent stationary phase (Figures 2A, B). The *recN* mutant did not compact its nucleoid after the addition of MMC and filamented extensively (Supplementary Figure 1). The amount of DAPI fluorescence per nucleoid decreased significantly during the recovery period suggesting that replication might be stopped and DNA degradation active up to 150 min after MMC wash. 5 h post-treatment, cells where still mainly forming filaments with diffuse nucleoids (Figures 2C, D). After BLM treatment, WT cells stopped dividing quickly (Supplementary Figure 1) and compacted their nucleoid (Figures 2E, F). Interestingly the mean DAPI fluorescence per nucleoid stayed high for 4 h after the treatment, suggesting that DNA compaction is prolonged in BLM compared to what we observed in MMC. In good agreement with CFU data, recovery was less efficient than in MMC. This was illustrated by many anucleated cells (21% in BLM compared to 0.3% in MMC, *P* = 0.00054) at time point 6 h and a large distribution of the average DAPI fluorescence in the nucleoid (Figures 2E, F). The *recN* mutant did not show nucleoid compaction, confirming that this property is linked to RecN and independent on the DNA lesions (Odsbu and Skarstad, 2014; Vickridge et al., 2017). Six hours post-treatment most cells did not yet recover a normal morphology (Supplementary Figure 1) with either diffuse or very compact small nucleoids (Figures 2G, H). Imaging results suggest that RecN activity is essential to induce the nucleoid compaction observable at early stages of the damage repair phase of MMC and BLM-induced lesions. Moreover, in the presence of BLM, the amount of DAPI per nucleoid of the WT cells remained high for a long period (up to 4 h), this might explain the high SCI frequencies observed on Figure 1D. Altogether, our results show different perturbations of nucleoid morphology and sister chromatid dynamics by the processing of MMC or BLM-induced lesions. Since RecN influence differs according to the drug, RecN might play different roles for the processing of the two drugs, perhaps linked to different partners.

### TIS analysis of RecN epistasis

**FIGURE 3:**
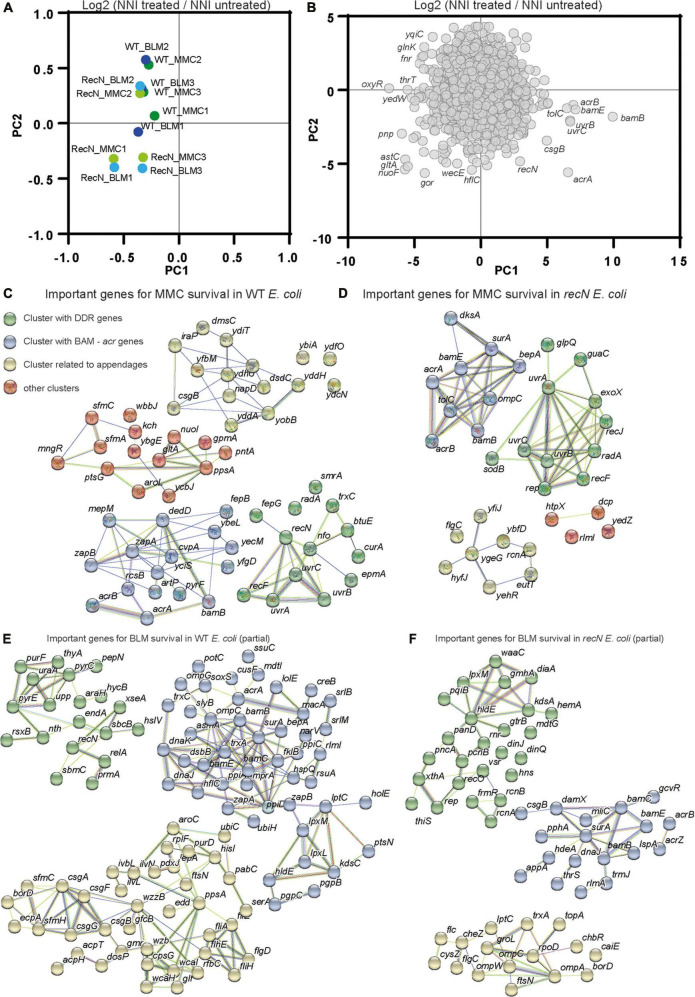
Transposon-insertion sequencing (TIS) analysis of the GSR associated with BLM and MMC tolerance. **(A)** Principal component analysis of TIS data. Fold changes (Log2 NNI treated/NNI untreated) were used to cluster conditions and replicates. **(B)** Principal component analysis of TIS data. TIS fold change in the different conditions and replicates was used to cluster genes. **(C)** Functional Kmeans clustering (https://string-db.org, medium confidence score) of important genes (Log2 FC _*MMC–*_*_*wt*_* < –2, *P*-value <0.1) for MMC survival in WT *E. coli*. **(D)** Functional Kmeans clustering of important genes for MMC survival in the *recN* strain. **(E)** Functional Kmeans clustering of important genes for BLM survival in WT *E. coli*. Only a portion of the genes was represented (63/123), the full list is available on Supplementary Table 1. **(F)** Functional Kmeans clustering of important genes for BLM survival in the *recN* strain. Only a portion of the genes was represented (52/95).

Transposon-insertion sequencing (TIS) methods, which combine genome-wide transposon mutagenesis with high-throughput sequencing allows the estimation of fitness contribution or essentiality of each genetic component in a bacterial genome. TIS gives the opportunity to decipher the pathways that led to WT and *recN* mutant survival in response to MMC and BLM toxicity and perhaps to enhance the characterization of its functioning for SC dynamics. TIS relies on fitness differences between Mariner Transposon insertion mutants. We expected to observe the effect of Mariner insertions leading to loss and gain of function in the in the WT and the *recN* strains. TIS analysis were performed in triplicates in identical conditions with three Mariner libraries, 1 million clones each, which were generated extemporaneously. Bacteria were submitted, or not, to a 20 min treatment with the drug, washed extensively and platted on LB (see section “Materials and methods”). The next day, we collected about 1 million colonies for each sample and extracted genomic DNA. We mapped about 10 million insertions in the genome of *E. coli*. The Normalized Number of Insertions (NIN) was measured for each gene of the genome for each replicate and condition. Component analysis revealed a significant dispersion of the replicates (Supplementary Figure 2A). We observed that considering the NIN log ratio of treated over untreated samples for each Mariner library reduced the dispersion (Figure 3A). This suggests that heterogeneous transposition efficiencies is at the origin of a large part of the difference between replicates. Applied to genes, PCA revealed that few genes explain the differences between the samples (Figure 3B). Among them were *recN*, the *uvrABC* genes involved in NER, genes from the BAM complex, the *acrAB-tolC* efflux pump, *oxyR* the regulator of oxidative stress response and metabolic genes. We performed a functional enrichment analysis of protein-protein interaction networks with the String tool^1^ on genes presenting a significant Fold Change (FC), mean Log2 FC <−1 and *P*-value <0.1, Supplementary Figure 2B and Supplementary Table 1. It revealed clusters of genes important for survival in the four conditions (WT + MMC, WT + BLM, *recN* + MMC, *recN* + BLM). First, both WT and *recN* mutant’s survival to MMC and BLM depended on a cluster of genes linked to the BAM complex (Figures 3C–F). This reflects presumably the requirement for the insertion in the outer membrane of the TolC β barrel protein that functions as a multi-drug efflux pump in coordination with AcrA (Log2 FC_*MMC–wt*_ = −3.6, Pval_*MMC–wt*_ = 0.09) and AcrB (Log2 FC_*MMC–wt*_ = −2.8, Pval_*MMC–wt*_ = 0.06). In addition to AcrAB, TolC cooperates with other efflux systems MdtA, MdtB, MdtC, MdtEF, EmrABC, EmrYK, AcrE, and AcrF which mutants were less detrimental for survival in these conditions (Supplementary Table 1). These observations suggest that although WT cells and *recN* cells can repair MMC and BLM lesions they cannot do it if the intracellular concentration of the drug is too high. We confirmed this result directly with the *acrA* deletion mutant that was unable to induce SOS nor *ParaBAD* promoters in the presence of MMC and showed a very poor viability (Supplementary Figure 4A). The functional clusters formed around the BAM complex also presented genes encoding proteins of diverse functions. Most of them were not common to the four conditions, suggesting that their role might be specific to the drug or the *recN* vs. wt backgrounds. We did not analyze them further.

The second cluster of genes detected in the TIS data reflects DNA Damage Repair (DDR). Interestingly this cluster presented diverse members according to the conditions (Figures 3C–F). In the presence of MMC, viability of the WT strain depended on the presence of functional *recN*, *recF*, *uvrABC*, *radA*, *smrA*, and *nfo* genes. In the presence of BLM, this cluster changed, it contained *recN*, *sbcB*, *nth*, *endA*, *XseA*, and *sbmC*. The absence of *recN* did not change the dependence on *uvrABC*, *recF*, and *radA* but revealed new important genes: *exoX* and *rep* for MMC survival. The absence of RecN profoundly modified the gene set required for survival to BLM, it included *recO*, *rep*, *vsr*, and *xthA*.

We did not analyze further the other clusters revealed by String but we noticed the presence of genes involved in the stringent response (*dksA* and *relA*), cell division (*zapA*, *zapB*, *ftsN*, and *damX*), oxidative phosphorylation/oxidative stress (*nuoI*, *soxR*, and *trxC*) or extracellular appendages (*csgABGF*, *sfmAC*, and *flgC*) suggesting that other cellular pathways were involved in genotoxic drug tolerance.

### TIS validation

To control TIS reliability, we constructed mutants of 20 selected genes in WT MG1655. We picked genes that presented TIS FC differences in MMC and BLM. First, we analyzed the gene set as a whole (Supplementary Figure 4B). The CFU of the mutants challenged with a 20 min MMC treatment correlated with TIS results (Slope of the linear regression = 0.97, *R*^2^ = 0.87). The CFU of the mutants challenged with a 40 min treatment followed a linear regression with a slope = 0.50 and *R*^2^ = 0.80. Therefore, TIS data adequately revealed colony formation of MG1655 treated for identical times. Results for the BLM treatment were less convincing. At 20 min the slope for the linear regression was 0.33 with a *R*^2^ = 0.38 and at 40 min the slope = 0.25 with a *R*^2^ = 0.51. Spearman correlation coefficients, 0.53 and 0.65, respectively, at 20 and 40 min indicated that overall TIS and CFU were correlated although data did not follow a monotonous trend. CFU and TIS data of several individual genes correlated very well. For instance, both TIS and CFU showed the importance of UvrA in the presence of MMC but not BLM. In addition, the viability loss of *acrA*, *pyrE*, *borD*, and *hflC* deletion mutants after BLM treatment was comparable to the TIS results. Interestingly, we also observed gain of function of some Mariner insertions in the *nuoA*, *fruK*, and *ybfE* genes and their corresponding deletions. Altogether, these results validated the TIS analysis as a good predictive tool for genes involved in DNA damage tolerance and repair.

### The DDR pathways at play for MMC lesions repair in WT and *recN* strains

**FIGURE 4:**
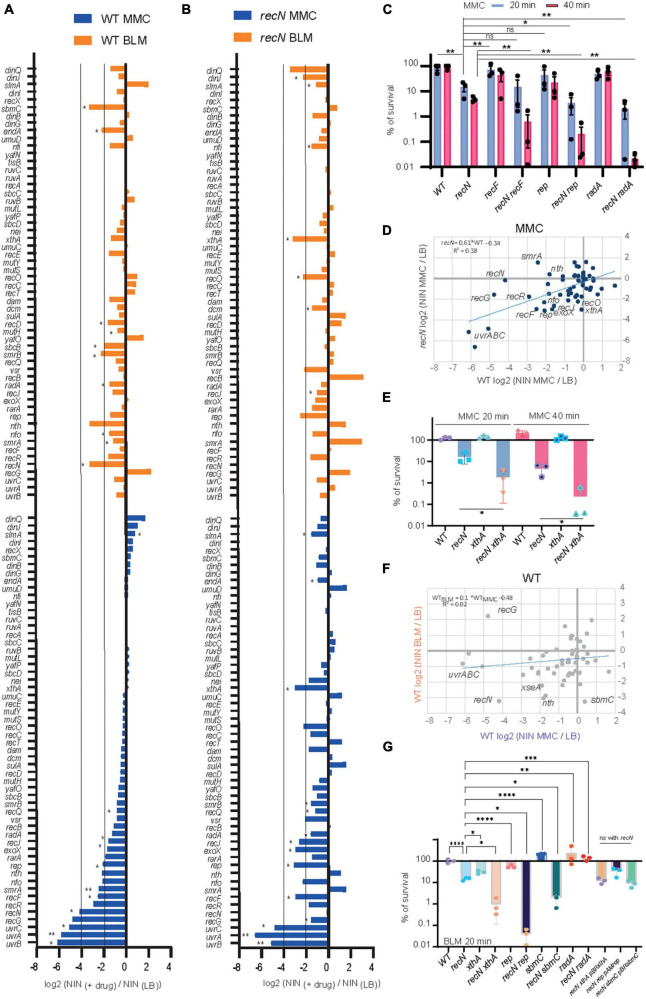
Transposon-insertion sequencing (TIS) analysis of the DDR genes involved in BLM and MMC tolerance. **(A)** TIS results in the presence of MMC or BLM for the subset of *E. coli* DDR genes in the WT strain. Results are average of three replicates and analyzed with multiple paired *t*-test *<0.1; **<0.05 **(B)** same as A for the *recN* strain. **(C)** CFU analysis of the WT, *recN*, *recF*, *rep*, and *radA* single and double mutants in the presence of MMC. Anova statistical test *<0.05, **<0.005 **(D)** comparison of the TIS results in MMC for the WT (Log2 FC _MMC–WT_) and *recN* strain (Log2 FC _*MMC–*_*_*recN*_*). **(E)** CFU analysis of the WT, *recN*, *xthA*, and *recN xthA* mutants in the presence of MMC. Anova statistical test *<0.05. **(F)** Comparison of the TIS results in WT strain in MMC (Log2 FC _*MMC–*_*_*WT*_*) and BLM (Log2 FC _*BLM–*_*_*wt*_*). **(G)** CFU analysis of the WT, *recN*, *xthA*, *recN xthA*, *rep*, *recN rep*, *sbmC*, *recN sbmC*, *radA*, and *recN radA* mutants in the presence of MMC. To reveal additive effects of the double mutants BLM at 0.5 μg/ml for 20 min was used. Anova statistical test *<0.05, ^**^<0.005, ^***^<0.0005, ^*⁣*⁣**^<0.00005.

To better characterize the DDR pathways involved in MMC and BLM repair, we observed TIS results for the whole gene set annotated for DNA repair (56 genes in Ecocyc, Figure 4). It is noteworthy that TIS is not adequate to study genes that present a strong growth disadvantage in the unchallenged condition such as *recA*, *ruvA,B,C*, and *priA,B* for example (Supplementary Table 1). After MMC challenge, the survival of the WT strains was dependent on UvrABC, RecG, RecN, RecF, RecR, SmrA, Rep, RecJ, RadA, and Vsr (Figure 4A). Mariner insertion mutants in *nfo* (Endonuclease IV), *nth* (Endonuclease III), *rarA* and *exoX* presented important mean reductions (Log2 FC_*MMC–wt*_ <−2) but with the larger deviations between replicates (Figure 4A). These observations suggested that different pathways cooperated in the GSR accompanying MMC-induced lesions: NER (UvrABC), homologous recombination (RecF, RecR, RecG, RecN, RadA, and RecJ), replication fork stalling (Rep) and perhaps BER (Endo III and IV). UvrABC, RecF, RecR, RecJ, Nfo, Rep, ExoX, and RadA were still required in the absence of RecN, suggesting the presence of different epistatic groups in the GSR (Figure 4B). To test this hypothesis, we performed CFU measurements of the single *recF*, *rep* and *radA* and double *recN recF*, *recN rep*, *recN radA* mutants (Figure 4C). Although single *recF*, *rep*, and *radA* mutants presented less viability defect than expected from TIS data, we observed significant viability decreases of each double mutant compared to *recN*. This confirmed that RecN and RecF, Rep or RadA belong to different epistasis groups.

Overall TIS results of the *WT* and *recN* strains correlated well (slope = 0.61, *R*^2^ = 0.4, Figure 4D). This suggests that although RecN absence changes viability and morphology of the cells, it does not imply massive changes in the GSR. Nevertheless, we observed a few interesting differences between the two strains. First, the inactivation of *nth*, encoding Endo III, was detrimental in the *WT* but slightly beneficial in the *recN* strain. Concomitantly, the selective pressure was much higher on *xthA* (EndoVI) and Nei (Endo VIII) when RecN was absent. Endo III and Endo VI, respectively, catalyze the first (glycosylase) and second (AP endonuclease) steps of the BER pathway of FappyA and Opyr (Daley et al., 2010; Prakash et al., 2012). CFU confirmed the synergic effect of *xthA* and *recN* mutants (Figure 4E). The second, interesting observation was that inactivation of *recG* was much less detrimental in the *recN* strain (Log2 FC_*MMC–recN*_ = −1.6) than in the *WT* (Log2 FC_*MMC–wt*_ = −4.8). This is in agreement with previous observation in *B. subtilis* (Sanchez et al., 2007) and suggests that in the absence of RecN alternative repair pathways are engaged. Altogether, our results suggest that survival to MMC toxicity requires processing by NER, homologous recombination and BER. The absence of RecN increases the pressure on the BER (XthA and Nei) and the processing of stalled replication (Rep) while it might concomitantly switch homologous recombination from a RecG dependent pathway to a less efficient RecF, RecR, RecJ, and RadA dependent pathway.

### The DDR pathways at play for BLM lesions repair in WT and *recN* strains

The GSR accompanying BLM-induced lesions was different from those accompanying MMC-induced lesions (Figures 4A, B, F). Only *recN*, and *nth* (EndoIII) were important for survival of the WT strain in both conditions. In addition, TIS revealed a role for SmrAB, EndA, RadA, SbcB (*xonA*), XthA, Nfi, and SbmC for the processing of BLM lesions in the WT strain. Obviously, we must also add RecA to this group (Figure 1A). According to the literature, these proteins contribute to HR, BER and replication restart pathways. We can split them into two groups according to their importance in the absence of RecN (Figures 4A, B). When RecN was absent, *radA*, *sbmC*, *sbcB*, *endA* and *nth* lost their importance (Figure 4B) suggesting that their activities were required in a RecN involving pathway(s). The presence of RadA in this group suggests that BLM-induced lesion are processed by an HR pathway that becomes dispensable or deleterious when RecN is absent. The observation that *recB* (Log2 FC _*BLM–*_*_*recN*_* = 3.13), *recC* (Log2 FC _*BLM–*_*_*recN*_* = 0.49) and *recD* (Log2 FC _*BLM–*_*_*recN*_* = 1.17) displayed positive FC in the absence of RecN confirmed this hypothesis. Reckless DNA degradation by RecBCD might explain the poor survival of the *recN* mutant to BLM. The presence of SbcB and Nth in the RecN dependent group, and XthA *and* Nfi in the RecN independent group suggest that BLM-induced lesions also require a processing by the BER pathway. In addition, the dependence on the Rep helicase (Log2 FC _*BLM–*_*_*recN*_* = −1.17 and Log2 FC _*BLM–*_*_*recN*_* = −3.17, Supplementary Table 1) suggests that replication forks are frequently stalled by BLM-induced lesions in the absence of RecN. We analyzed by CFU single *xthA*, *rep*, *sbmC* (a gyrase inhibitor) and *radA* mutants and double mutants combined with *recN* deletion (Figure 4G). In good agreement with the TIS data, the viability of the *xthA* mutant was significantly reduced compared to the WT strain and that of the *recN xthA* double mutant significantly reduced compared to the *recN* mutant. This additive effect suggest that XthA and RecN works in parallel pathways to repair BLM lesions. Results were more puzzling with the other mutants: the viability of the single *rep* and *sbmC* mutants was not significantly different from the WT strain but the double *recN rep* and *recN sbmC* mutants showed a strong synergic effect. Surprisingly, we observed that *radA* and *recN radA* mutants tolerated exquisitely BLM suggesting that an important part of lethality associated with the lack of RecN is the consequence of RadA activity. These observations differ from TIS results and suggest that TIS results with BLM should be taken cautiously and confirmed with conventional CFU assays.

### The involvement of NER differentiates MMC and BLM induced lesion repair

**FIGURE 5:**
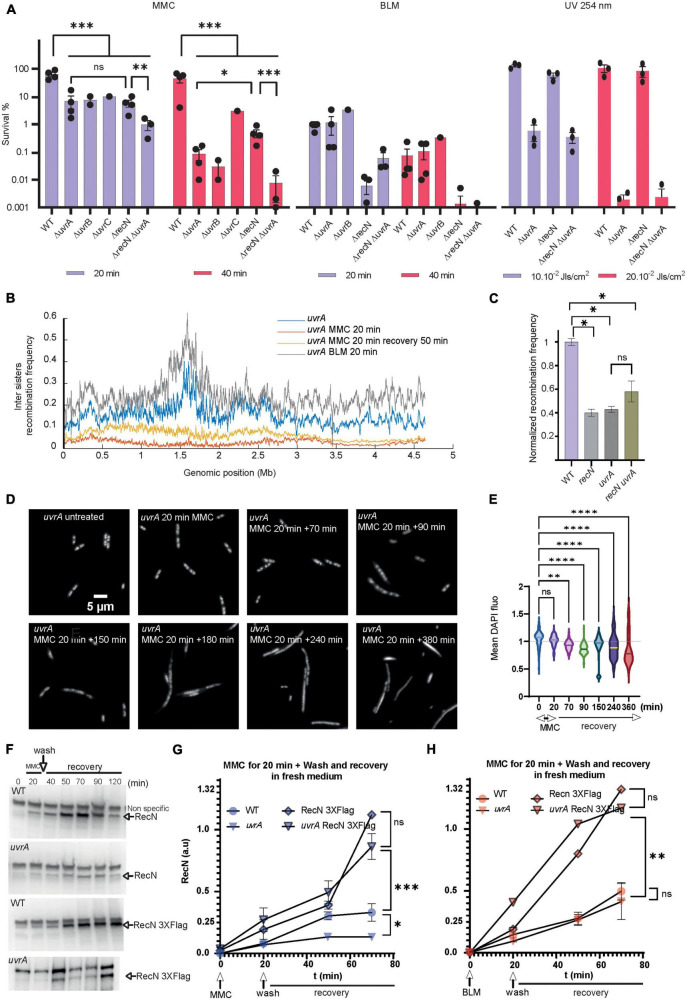
Role of the NER in the GSR associated with BLM and MMC lesions. **(A)** CFU analysis of the WT, *recN*, *uvrA*, *uvrB*, *uvrC* single, and double mutants in the presence of MMC, BLM, and UV irradiation. Anova statistical test *<0.05, ^**^<0.005, ^***^<0.0005, ^*⁣*⁣**^<0.00005. **(B)** Hi-SC2 profile of sister chromatid interactions in the *uvrA* mutants in the presence or absence of MMC and BLM. **(C)** Measure of inter sister locus recombination, *laclox* assay at the *aidB* locus (Vickridge et al., 2017), in the WT, *recN*, *uvrA*, and *recN uvrA* mutants. **(D)** Nucleoid imaging of the *uvrA* mutant during the recovery period of MMC injury. **(E)** Quantification of the mean DAPI fluorescence in the nucleoid area of *uvrA* mutant treated with MMC. Anova statistical test *<0.05, ^**^<0.005, ^***^<0.0005, ^*⁣*⁣**^<0.00005. **(F)** Western blot showing RecN and RecN 3XFlag amount in WT and *uvrA* cells during GSR recovery. **(G)** Quantification of RecN amount in 3 western blot experiments performed as in panel F in the WT and *uvrA* mutant during the recovery period of MMC injury. RecN amount was normalized to the amount of RNA polymerase β in each sample. **(H)** Measure of RecN and RecN 3X-flag turnover in the WT and *uvrA* mutant during the recovery period of BLM injury (*N* = 3 western blots performed in identical conditions).

Transposon-insertion sequencing (TIS) and CFU data showed that MMC and BLM induced lesions require both HR and BER pathways, with RecN playing an important role in the flux or the outcome of these processes. Differently, NER only participates in the GSR accompanying MMC (Figures 4, 5A). Surprisingly, we only observed a modest additive effect when we combined *recN* and *uvrA* deletions, suggesting that they might contribute to a common pathway (Figure 5A). This pathway differ from that involved in UV lesion repair that does not require RecN (Figure 5A). This led us to consider that NER could be responsible for the different sister chromatid dynamics that we observed in response to MMC and BLM-induced lesions. In agreement with CFU data, the *uvrA* mutant has no influence on SCI nor nucleoid compaction following BLM treatment (Figure 5B and Supplementary Figure 4A). Hi-SC2 showed a decrease of SCI in the *uvrA* mutant comparable to that of the *recN* mutant in MMC (RF = 2%; PC_WT–MMC20/uvrA–MMC20_ = 0.29, Figure 5B). L*aclox* assay (Lesterlin et al., 2012) at a single locus confirmed this observation and showed that combining *recN* and *uvrA* deletions has no additive effect on SCI loss (Figure 5C). Imaging showed a nucleoid decompaction in the *uvrA* mutant comparable to the *recN* mutant (Figures 5D, E). Thus, in the absence of NER activity RecN has no influence of sister chromatid dynamics and nucleoid morphology. RecN induction was slightly reduced in the presence of MMC in the *uvrA* mutant (Supplementary Figure 4B). However, RecN accumulation in the presence of MMC was unchanged (Figures 5E, F, time point 20 min), suggesting that RecN was produced in the absence of NER. Following MMC’s wash (time points 40 to 120 min), less RecN was accumulated in the *uvrA* mutant compared to the WT (Figures 5F, G). By contrast, no difference was observed between WT and *uvrA* strain after BLM’s wash (Figure 5H). Stabilizing RecN with a C-terminal Flag epitope restored the RecN amount in the *uvrA* strain to a level comparable to the WT. Those results suggested that the amount of RecN was controlled by its degradation in the recovery phase. Using *dnaCts* non-replicating cells, we observed that the amount of RecN is directly dependent on functional NER, confirming that NER is the main driver of RecN stability in cells treated with MMC (Supplementary Figure 4C). Altogether these results suggest that RecN is rapidly degraded in the absence of NER making its amount to drop below a level that allows sister chromatid interactions and optimal repair by HR.

### In the NER mutants, survival to MMC toxicity relies on alternative GSR

**FIGURE 6:**
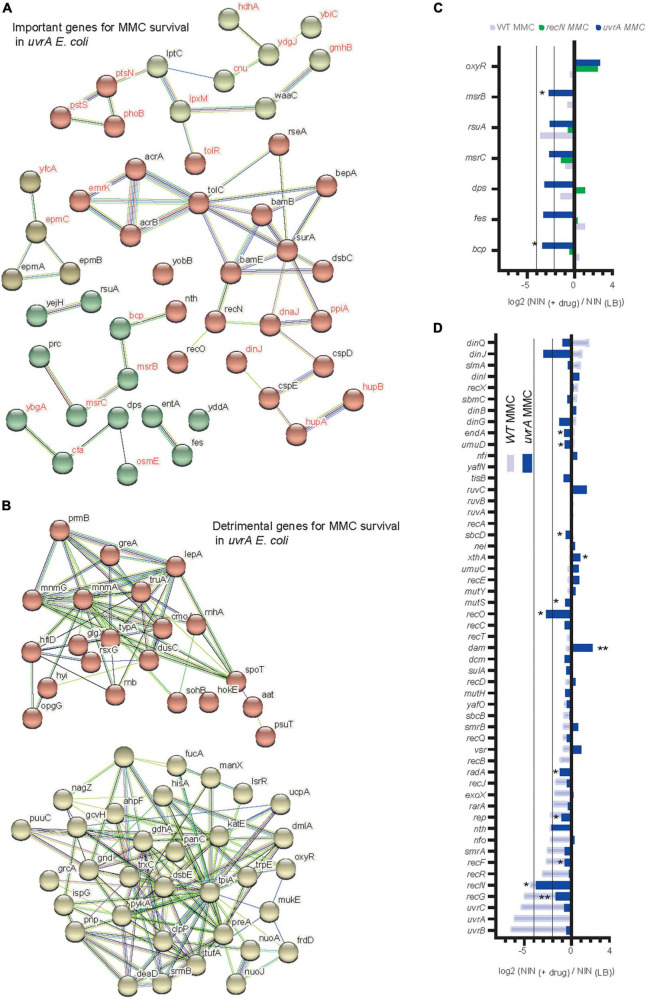
Transposon-insertion sequencing (TIS) analysis of alternative pathways to NER for MMC tolerance. **(A)** Functional Kmeans clustering of important genes for MMC survival in the *uvrA* strain. **(B)** Functional Kmeans clustering of the counter selected genes for MMC survival in the *uvrA* strain. **(C)** TIS results in the presence of MMC for a subset of genes involved in oxidative stress tolerance in the WT, *recN*, and *uvrA* strains. Results are average of three replicates and analyzed with multiple paired *t*-test *<0.1. **(D)** TIS results in the presence of MMC for the subset of *E. coli* DDR genes in the WT and *uvrA* strains. Results are average of three replicates and analyzed with multiple paired *t*-test *<0.1, **<0.05.

To decipher which remaining genetic pathways are important to allow a small number of cells to survive MMC treatment in the absence of NER, we applied TIS to the *uvrA* mutant. As observed for the WT and the *recN* strains, the AcrAB-TolC efflux system and its BAM assembly complex were crucial for survival (Figure 6A). Interestingly, we observed a new set of important genes for survival. Those genes are related to oxidative stress: *msrC* and *msrB* two methionine-(*R*)-sulfoxide reductases (Ezraty et al., 2005), *rsuA*–a pseudouridine synthase, which overexpression increases MIC for hypochlorous acid (Chen et al., 2021), *bcp*–a thiol peroxidase, *bcp* deletion mutants are hypersensitive to peroxides (Mishra and Imlay, 2012), fes–the enterobactin esterase, fes mutant is more sensitive to hydrogen peroxide (Peralta et al., 2016) and *dps*–a stationary phase DNA binding protein that sequesters iron to prevent DNA damage (Calhoun and Kwon, 2011; Figures 6B, C). This relationship with oxidative stress was also illustrated by the strong selection for Mariner insertion inside *oxyR*, which codes for the main regulator of the oxidative stress response in *E. coli* and the *nuo* genes, which code for non-essential proteins of the respiratory chain (Figure 6B). Another pathway emerged from the TIS analysis: enzymes involved in RNA metabolism and turnover (*greA*, *rnhA*, *rnb*, *pnp*, *deaD*, and *srmB*). Mariner insertions in all of these genes are positively selected in the presence of MMC (Figure 6B). Finally we observed that among DDR genes only RecN, *nth*, and *recO* kept a role in the *uvrA* mutant (Figure 6D) while insertions in *clpP* were positively selected (Supplementary Figure 4D), suggesting that an alternative repair pathway requiring the maximum possible amount of RecN was still active in these conditions and allowed a minimal survival of some cells. Altogether, our results suggest a positioning of RecN at the crossroad of multiple repair pathways, playing a role in activating HR and facilitating the functioning of the BER pathway, stimulated by NER to promote sister chromatid and nucleoid remodeling and perhaps functioning in a yet uncharacterized pathway with RecO but not RecF and RecR.

## Discussion

### TIS is adequate to survey epistasis at the whole genome level

To characterize the epistasis relationship between RecN and other genes of the *E. coli* genome we used Transposon insertion sequencing (TIS). TIS is a powerful approach that can be extensively applied to the genome-wide definition of loci that are required for bacterial growth under diverse conditions (Elhenawy et al., 2021; Prudent et al., 2021). However, to our knowledge, TIS has not yet been used to compare DDR and GSR in response to treatment with different genotoxic compounds. Experimental design choices and stochastic biological processes can heavily influence the results of TIS experiments and affect downstream statistical analysis (Chao et al., 2016). The power of any TIS study is greatly influenced by the complexity of the initial library generated and the maintenance of this complexity during selection. In *E. coli*, the DDR is usually analyzed with CFU that reflect very low survival rates of WT or mutant strains (Picksley et al., 1984; Michel et al., 2000). In such conditions bottleneck effects are unavoidable and might bias TIS statistical analysis. To limit these biases we created a library with 2 million independent clones and worked with short exposure to low doses of MMC and BLM. We constructed mutants of more than 20 TIS hits and tested them by CFU. Overall TIS and CFU data correlated well (Spearman correlation coefficient ranging from 0.53 to 0.92, Supplementary Figure 3B). For the stronger TIS hits (*acrA* and *acrB*, *uvrA* or *recN*) the correlation between TIS data and CFU was very good (Supplementary Figure 3B). However, for genes that presented a small fitness loss (> Log2 FC = −1) the correlation with CFU was less satisfactory. This suggests that according to the experimental conditions, different sets of genes might be revealed by TIS. Therefore, in future experiments, dose response or duration of the treatment could be tuned to reveal different faces of the GSR.

### TIS reveals the GSR

**FIGURE 7:**
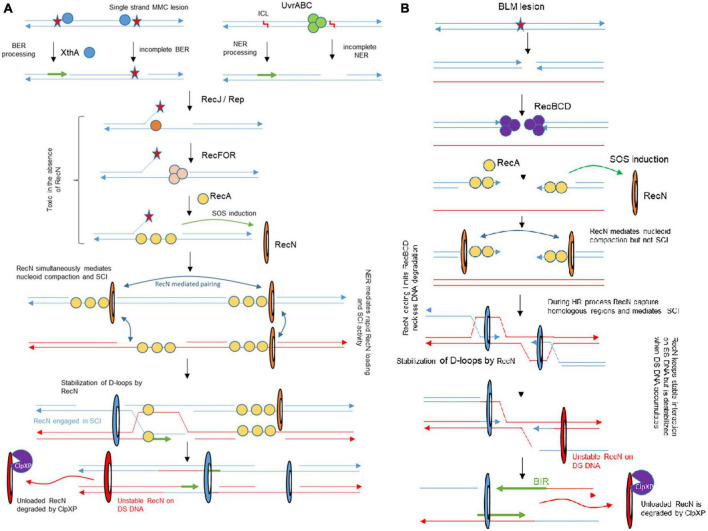
Integrated model for genotoxic stress response accompanying MMC and BLM induced lesions and its impact on SCI. **(A)** The NER and BER pathway are involved in MMC tolerance. Our results show that early processing by NER or BER are required for RecN activity, presumably for its loading. In the absence of RecN, BER intermediates become toxic for the cell. RecN plays multiple roles in the following steps of the process, it mediates nucleoid compaction, SCI and facilitates RecA mediated D-loops. When SS-SNA disappear, RecN dissociates from the DNA and is degraded. **(B)** In the presence of BLM, the RecBCD pathway allows SOS induction and RecN loading. RecN contributes to limit RecBCD reckless DNA degradation. Although RecN impact on nucleoid compaction manifests as early as the one observed in the presence of MMC, its influence on SCI is delayed. We only observed RecN influence on SCI when BLM was washed, at a time when repair by HR should already be engaged.

Although the genotoxic activities of MMC and BLM were characterized several decades ago (Suzuki and Kilgore, 1967; Burger et al., 1981) and their usage in cancer therapy initiated in the 80’s (Garewal, 1988), the Genotoxic Stress Response (GSR) associated with those antibiotics in *E. coli*, and other bacteria, remains incompletely characterized. Although, screens were carried out to identify genes conferring a hypersensitivity to MMC and BLM (Becket et al., 2010), these studies only focused on WT *E. coli* and therefore did not question epistasis relationships. The originality of our study was to perform TIS in three different genetic backgrounds (WT, *recN*, and *uvrA* mutants) challenged with two drugs. This strategy allows us to propose an integrated model of the GSR that includes equilibrium changes in the different contexts (Figure 7).

### RecN plays major roles in nucleoid compaction and sister chromatid cohesion

Previous literature indicated that three DDR proteins were commonly required for the survival to BLM and MMC toxicity: RecA, RecN, and RecG; among them RecN activities were the less characterized. Previous studies showed that RecN, an SMC protein, works to maintain two DNA strands in close contact (Pellegrino et al., 2012; Vickridge et al., 2017; Keyamura and Hishida, 2019). It was therefore proposed that RecN tethers DNA ends or maintains homologous or non-homologous regions in contact as cohesins do. To decipher the role(s) of RecN during the repair of MMC and BLM we used a genome wide SCI assay (Figure 1C). It revealed different sister chromatid dynamics associated with RecN activity on chromosomes altered by MMC-induced lesions compared to BLM-induced lesions. We observed that RecN contributes to maintaining SCI along the whole genome in the presence of MMC but it plays no role during the recovery period, after the wash of the drug. By contrast, RecN did not influence initial SCI in the presence of BLM but played an important role during the recovery period to produce extra interactions between SC. Surprisingly this difference did not correlate with nucleoid compaction which is entirely under the control of RecN in MMC and BLM. These observations suggest that these two events, Sister chromatid cohesion and nucleoid compaction, might not reflect the same RecN activity. A partnership between RecN and NER in the GSR accompanying MMC-induced lesion might explain this difference. We propose that MMC-induced lesions are rapidly processed by NER in a way that allows massive loading of RecN at multiple places on the chromosome. Since RecN prefers single strand DNA for binding it is tempting to propose that in MMC conditions many NER processing events are not complete and generate single strand DNA (Weng et al., 2010). The observation that in the absence of DNA replication NER was required to induce RecN and presumably every SOS gene, confirmed this hypothesis (Supplementary Figure 3A). Once loaded, RecN prevents segregation of sister loci and provokes nucleoid compaction. In the recovery phase, the progressive repair of single stranded regions shall favor RecN unloading and its subsequent degradation.

In the presence of BLM, RecN activity was only visible later during the recovery phase. One possible explanation for this observation is that adequate single strand DNA substrates are not immediately available for RecN. BLM lesions produce double strand breaks that will be processed by RecBCD and covered by SSB and RecA. Since RecN is only induced at this stage, we speculate that it may function only after the initial homology search and D loop formation by RecA. At this timing, its loading on SS DNA could stabilize D loops as observed *in vitro* (Reyes et al., 2010). Replication on these RecN loaded substrates, (from *oriC*, from repair intermediates or from the ectopic regions) might explain the important increase of SCI that we observed in the recovery phase.

### RecN is at the crossing point between different repair strategies

Combining Hi-SC2, TIS, imaging and viability assays we can propose an integrated picture of the GSR accompanying MMC and BLM-induced lesions (Figure 7). The strong requirement for a preprocessing of MMC-induced lesions to stabilize RecN and promote SCI and presumably HR was unexpected; although it is well appreciated that HR and NER work in coordination (Ide et al., 2008; Li and Heyer, 2008), the strong requirement of NER processing for the activity of an HR protein partner has not yet been described in *E. coli*. Recently, a similar partnership was described for NER and TLS in non-replicating *C. crescentus* bacterium (Joseph et al., 2021), such partnerships might therefore be more frequent than expected during GSR. Future genome wide epistasis analysis with TIS might help to reveal their nature and complexity. Our MMC results suggest that RecN loads frequently on SS gaps generated by imperfect NER or BER processing. The strong dependency on RecN for the repair of DSB generated by endonucleases (Meddows et al., 2005) suggests that it is also active on RecBCD processed events, although in the presence of MMC such events seem to be rare. RecN importance for BER deficient cells was unexpected. It has been proposed that the products of Glycosylases (Nei and Nth) activity on damaged pyrimidines can be toxic when their processing by AP endonucleases (XthA or Nfo) and Polymerase and ligase is not fast enough (Spek et al., 2002; Çaglayan and Wilson, 2017). RecN therefore may be critical to process these intermediates. Finally, RecN may also participate to late steps of the GSR. Because we observed its influence on SCI late in the recovery process of BLM-injured DNA, we suspect that it corresponds to its loading on SS gaps generated during HR or Break Induced Replication. Altogether our results show RecN influence in multiple repair pathways, playing a role in activating HR and facilitating the functioning of the BER pathway and stimulated by NER to promote sister chromatid and nucleoid remodeling. Therefore RecN plays a pivotal role in the GSR strategy.

## Materials and methods

### Strains and plasmids

All strain and plasmids used in this study are described in Supplementary Tables 2, 3. Studied strains derived from the wild-type MG1655. Strains constructions were done by P1 transduction or recombineering.

### Growth conditions

Experiments were performed in LB (1% de bacto-tryptone, 0.5% yeast extract, 1% de NaCl, pH 7.5) or on LB-agar (LB + 1.5% de bacto-agar). Viability assays were performed as described (Vickridge et al., 2017). Mitomycin C (Sigma-Aldrich and reference M0503) was used at 5 μg/mL. We noticed that toxicity of BLM (Bleomycin Sulfate, Sigma-Aldrich, and reference B1141000) varies significantly according to the lot number. Therefore, we adapted BLM concentration (0.1 to 0 2 μg/mL) and always included the WT and *recN* mutant in each assay as references. Figures and statistical analysis were produced with Prism (Graphpad).

### Expression assays

An overnight culture of strains containing the plasmid p*PrecN*-GFP or pFCCGI (*ParaBAD*-GFP) was diluted 1/200 in LB and grow until OD_600 nm:_ 0.2 at 37° in a Tecan Spark plate reader. BLM or MMC was added at concentrations of 1 μg/ml or 5 μg/ml, respectively, with 0.1% arabinose when required. Western blot were performed as described (Espeli et al., 2003). Anti RecN polyclonal antibody (IgG) was produced in rabbit (Eurogenetec) immunogenized with purified RecN. HIS14-SUMObd-GGGGGG-RecN bound on Ni-NTA column and GG-RecN was eluted after bdSENP1 cleavage. Primary antibodies were diluted 1/1 000, secondary antibody at 1/5 000. The anti-RNA-pol Beta antibody coupled to the HRP enzyme (OZYME) was added simultaneously at a 1/20 000 dilution. RecN amount are expressed as ratio with the RNA polymerase β signal.

### Transposon insertion sequencing (TIS)

Transposon insertion sequencing was performed as described (Yamaichi and Dörr, 2017) Mariner transposon was conjugated from *E. coli* SM10 MFD-*pir* carrying pSC189-Mariner, with an *E. coli* MG1655 strain. About 2 million independent clones, were plated on 10 20 cm × 20 cm LB agar plates supplemented with Kanamycin and Diaminopimelic acid (DAP) when required. The next day, colonies were recovered in 30 mL of LB. A culture of the Mariner library was set with a starting OD_600 *nm*_of 0.03 in 45 ml LB. The culture was grown at 30°C with agitation (150 rpm) until OD_600 nm_ 0.15. When required the genotoxic agent (MMC 5 μg/ml or BLM 0.5 or 0 1 μg/ml) was added for 20 min. Cultured were immediately washed twice in fresh LB and cells were spread to 20 cm × 20 cm LB agar plates at 30°C. According to the drug and mutant considered different dilutions were used to reach the target number of colonies. Since BLM toxicity varies according to the lot number, we performed preliminary tests to adapt TIS protocol to these changes. The next day, ≈ 2 million colonies were recovered from each condition in 30 mL of LB and genomic DNA extracted. The sequencing libraries were constructed as described (Yamaichi and Dörr, 2017). Sequencing was perform on a MiSeq Illumina system at the Imagif facility. FastQ files were processed with Tn-Seq explorer (Solaimanpour et al., 2015) with the Bowtie2 parameters local and very sensitive. The number of insertions per gene was normalized according to the number of TA dinucleotides per gene and then normalized by the total number of insertions in the sample to get the Normalized Number of Insertions (NIN). Genes collecting more than 5000 insertions were considered as Mariner host spot and excluded from the analysis. Data are deposited on GEO GSE228146.

### Hi-SC2

Hi-SC2 was performed in a similar way to Espinosa et al. (2020a). The *loxP* sites were introduced by conjugation and transposition. The conjugation were performed between *E. coli* β-2163 pSWT23 (Demarre et al., 2005; van Opijnen et al., 2009) and *E. coli* MG1655 carrying the recombination system OE2358 (WT), OE2360 (*recN*), and OE2361 (*uvrA*) at the *lacZ* site. Transposition library was obtained with the TIS protocol in presence of DAP (0.6 mM) and IPTG (0.1 mM) to repress Cre. One millions of colonies with independent LoxP cassette insertions were flash frozen at −80°C with 50% glycerol. Aliquots of the transposition library were placed on ice for thawing. Cells were grown to exponential growth phase (OD_600_: 0.2) from 10^8^ cells diluted in 100 ml LB with IPTG (OD_600_: 0.05). Then 1 ml of culture is centrifuged and transferred into 40 ml of new culture medium without IPTG at 30°C under agitation 180 rpm. After 90 min, when OD_600_: 0.1 was reached, the genotoxic agent was added for 20 min. Three washes in LB were performed and either arabinose (0.1%) was added immediately for 60 min or after 50 min of recovery time in LB. Cells were harvested and genomic DNA extracted and processed for sequencing as described (Espinosa et al., 2020a). Data were processed with Matlab scripts (Espinosa et al., 2020b).

### Microscopy

An overnight culture was diluted 1/200 in LB. The cells were grown to an OD_600 nm:_ 0.1 at 37°C, genotoxic agent was added for 20 min and washed three times in LB to allow recovery. Sample were fixed at the indicated time, stained with DAPI and observed on 1% agarose melted in PBS. Imaging was performed on a Zeiss Axio Observer 7 with 63X PL-APO, NA 1.40 objective and a sCMOS (Hamamatsu Flash 4) camera. The acquisition was done with ZEN software and the processing and analysis of the images with Fiji software and its application MicrobeJ (Ducret et al., 2016). Figures and statistical analysis were produced with Prism (Graphpad).

## Data availability statement

The original contributions presented in this study are included in the article/Supplementary material, further inquiries can be directed to the corresponding author.

## Author contributions

AC designed, performed and analyzed the experiments, constructed the tools, and prepared the figures. EE and PZ designed, performed and analyzed the experiments. PS designed and constructed the tools. NQ constructed the tools and performed the experiments. EV designed and supervised the experiments. MM designed, performed and analyzed the experiments, and acquired funding. FXB designed and supervised the experiments, interpreted the results, acquired funding, and wrote the manuscript. OE designed, analyzed and supervised the experiments, interpreted the results, acquired funding, and wrote the manuscript. All authors contributed to the article and approved the submitted version.

## Footnotes

1
https://string-db.org/

## Supplementary material

Supplementary Figure 1 Measure of cell area (Phase contrast) during genotoxic treatment (MMC and BLM) and recovery period in WT **(A,C)** and *recN* mutant **(B,D)**. N ≈ 400, statistical significance was evaluated by Anova with Türkiye multiple comparison test of the mean cell area (*<0.033, ^**^<0.0021, ^***^<0.0002, ^****^<0.0001).

Supplementary Figure 2 **(A)** Principal component analysis of TIS data. Normalized Insertion Numbers (NIN) were used to compare conditions and replicates. **(B)** Volcano plot of the mean Log2 FC of TIS replicates and its *P*-value. Selected genes for the String clustering analysis are colored in orange.

Supplementary Figure 3 **(A)** Induction of the *recN* and *araBAD* promoters in WT and *acrA* strain in the presence of MMC (5 μg/mL) or BLM (0.1 μg/mL). **(B)** Comparison of TIS results expressed as mean Log2 FC (NIN treated/NIN untreated) and CFU expressed as mean Log2 FC of the number of colonies in the treated and untreated conditions. Mutants available in the Keio collection were transduced in the WT MG1655 strain and tested after MMC (μg/mL) and BLM (1 μg/mL) treatment. Linear regression (black lines) with 95% confidence intervals (dashed lines) are presented. Pearson (*R*^2^) and Sperman coefficient were measured to evaluate de correlation between TIS and CFU data.

Supplementary Figure 4 **(A)** Imaging of the nucleoid of the uvrA mutant treated with BLM for 20 min, washed and allowed for recovery. **(B)** Induction of the *recN* and *araBAD* promoter in WT and *uvrA* strain in the presence of MMC or BLM. **(C)** RecN production measured by western blot in replicating and non-replicating WT and *uvrA* mutant cells. To generate non-replicating cells, initiation of replication of *dnaCts* cells was blocked for 90 min at 40°C before addition of MMC. **(D)** TIS results for the *clpA*, *clpP*, and *clpX* genes in the WT, *recN* and *uvrA* mutants. Data are expressed as the difference between the mean NIN in the treated condition and the mean NIN in the untreated conditions. Multiple paired *t*-test *<0.1; ^**^<0.05.
