# Supplementary material for: The SMC-like RecN protein is at the crossroads of several genotoxic stress responses in Escherichia coli
Source: Front Microbiol. 2023 Apr 24;14:1146496. doi: 10.3389/fmicb.2023.1146496 (PMC10165496; doi:10.3389/fmicb.2023.1146496)
Supplement: Supplementary file 2 [file Table_2.DOCX]

**Supplementary Table 2**

| OE# | Genetic background | Genotype | Reference |
| --- | --- | --- | --- |
| 1 | MG1655 | *E. coli* K12 F- lambda- ilvG- rfb-50 rph-1 | Espeli lab stock |
| 2 | MG1656 | *MG1655 ΔlacMlul* | Espeli lab stock |
| 9 | MG1655 | *dnaC^TS^ (dnac2 ::Tc)* | Espeli lab stock |
| 480 | MG1656 | MG1656 *aidB ::LacLoxP frt* | (Lesterlin *et al*, 2012) |
| 486 | MG1655 | *∆recN::kan* | (Vickridge *et al*, 2017) |
| 256 | MG1655 | *∆recN:: frt::cm::frt* | (Vickridge *et al*, 2017) |
| 1184 | MG1655 | *ΔrecA* | (Vickridge *et al*, 2017) |
| 1595 | JJC267 | AB1157 uvrA6 malF::Tn10 tetR | Gift from B. Michel |
| 1597 | JJC2044 | JJC40 *∆uvrA::cm* | Gift from B. Michel |
| 1601 | JJC2162 | JJC40 *radA::kan* | Gift from B. Michel |
| 1629 | MG1655 | *∆uvrA::cm* (JJC 2044) | This work |
| 1632 | MG1655 | *∆radA::kan* P1 transduction from JJC 2162 | This work |
| 1633 | MG1655 | *∆rec*N *∆uvrA::Cm* P1 transduction from JJC 2044 | This work |
| 1636 | MG1655 | *∆recN* *∆radA::Kan* P1 transduction from JJC2162 | This work |
| 1637 | MG1656 | *aidB ::LacLoxP* *∆uvrA::Cm* P1 transduction from JJC 2044 | This work |
| 1641 | MG1656 | *aidB ::LacLoxP* *∆recN ∆uvrA::Cam* P1 transduction from JJC 2044 | This work |
| 1779 | MG1655 | RecN (LGGGGSRAA)-3XFlag *frt-cm-frt* | This work |
| 1780 | MG1655 | *dnaC^TS^* RecN (LGGGGSRAA)-3XFlag *frt-cm-frt* | This work |
| 1969 | MG1655 | *dnaC^TS^* RecN (LGGGGSRAA)-3XFlag *frt* | This work |
| 1972 | MG1655 | *dnaC^TS^* *∆uvrA::cm* RecN (LGGGGSRAA)-3XFlag *frt* | This work |
| 2357 | MG1655 | *lacZ:: cre frt_cm_frt* | Gift from Yoshi Yamaichi |
| 2358 | MG1655 | *lacZ:: cre frt* | This work |
| 2360 | MG1655 | *∆recN::Kan l*acZ:: Cre frt | This work |
| 2362 | MG1655 | *∆uvrA::cm l*acZ:: Cre frt | This work |
| 1815 | MG1655 | *∆recN ∆radA::Kan* | This work |
| 1843 | MG1655 | RecN (LGGGGSRAA)-3XFlag  *frt* | This work |
| 2217 | DY330 | *ΔradA::cm* | This work |
| 2230 | MG1655 | *ΔrecN frt* *ΔradA::cm* | This work |
| 1075 | MG1655 | *ΔsbmC::cm* | This work |
| 1076 | MG1655 | *ΔrecN* *frt* *ΔsbmC::cm* | This work |
| 2620 | MG1655 | *ΔnuoA ::kan* P1 transduction from KEIO collection | This work |
| 2622 | MG1655 | *ΔborD::kan* P1 transduction from KEIO collection | This work |
| 2623 | MG1655 | *ΔdinI::kan* P1 transduction from KEIO collection | This work |
| 2625 | MG1655 | *ΔnuoK::kan* P1 transduction from KEIO collection | This work |
| 2626 | MG1655 | *ΔamiA::kan* P1 transduction from KEIO collection | This work |
| 2635 | MG1655 | *ΔrecF::kan* P1 transduction from KEIO collection | This work |
| 2665 | MG1655 | *ΔrecN ΔrecF::kan* P1 transduction from KEIO collection | This work |
| 2636 | MG1655 | *ΔhslU::kan* P1 transduction from KEIO collection | This work |
| 2637 | MG1655 | *ΔhslV::kan* P1 transduction from KEIO collection | This work |
| 2638 | MG1655 | *ΔacrA::kan* P1 transduction from KEIO collection | This work |
| 2639 | MG1655 | *ΔyceH::kan* P1 transduction from KEIO collection | This work |
| 2640 | MG1655 | *ΔdadX::kan* P1 transduction from KEIO collection | This work |
| 2641 | MG1655 | *ΔyniA::kan* P1 transduction from KEIO collection | This work |
| 2642 | MG1655 | *ΔydjM::kan* P1 transduction from KEIO collection | This work |
| 2645 | MG1655 | *Δwzb::kan* P1 transduction from KEIO collection | This work |
| 2646 | MG1655 | *ΔyejH::kan* P1 transduction from KEIO collection | This work |
| 2647 | MG1655 | *ΔnuoI::kan* P1 transduction from KEIO collection | This work |
| 2648 | MG1655 | *ΔyfcJ::kan* P1 transduction from KEIO collection | This work |
| 2649 | MG1655 | *ΔpyrE::kan* P1 transduction from KEIO collection | This work |
| 2650 | MG1655 | *Δrep::kan* P1 transduction from KEIO collection | This work |
| 2680 | MG1655 | *ΔrecN Δrep::kan* P1 transduction from KEIO collection | This work |
| 2651 | MG1655 | *ΔpfkA::kan* P1 transduction from KEIO collection | This work |
| 2652 | MG1655 | *ΔhflK::kan* P1 transduction from KEIO collection | This work |
| 2653 | MG1655 | *ΔhflC::kan* P1 transduction from KEIO collection | This work |
| 2654 | MG1655 | *ΔpurA::kan* P1 transduction from KEIO collection | This work |
| 2655 | MG1655 | *ΔyceK::kan* P1 transduction from KEIO collection | This work |
| 2656 | MG1655 | *ΔpaaK::kan* P1 transduction from KEIO collection | This work |
| 2657 | MG1655 | *ΔybfE::kan* P1 transduction from KEIO collection | This work |
