# Supplementary material for: The SMC-like RecN protein is at the crossroads of several genotoxic stress responses in Escherichia coli
Source: Front Microbiol. 2023 Apr 24;14:1146496. doi: 10.3389/fmicb.2023.1146496 (PMC10165496; doi:10.3389/fmicb.2023.1146496)
Supplement: Supplementary file 3 [file Table_3.DOCX]

## Supplementary table 3

| Plasmides | Antibiotic resistance | Références |
| --- | --- | --- |
| pSC-189 DMF-Mariner | Kan + DAP | (Yamaichi & Dörr, 2017) |
| pCre | Spec | (Lesterlin et al. 2012) |
| pFCGi | Amp | PBAB-GFP PrpsM-mCherry (Helaine *et al*, 2014) |
| pPRecN_GFP | Spec | This work |
| pETHIS14-SUMO-GGGGGG-RecN | Amp | This work |
| pBR322 XthA | Kan | pBR322inc3 containing the region from argM to ynjA. This work |
| pBR322 SbmC | Kan | pBR322inc3 containing sbmC with its promoter. This work |
| pAMrep |  | Gift from Benedicte Michel |
