# Supplementary figures and images for: The SMC-like RecN protein is at the crossroads of several genotoxic stress responses in Escherichia coli
Source: Front Microbiol. 2023 Apr 24;14:1146496. doi: 10.3389/fmicb.2023.1146496 (PMC10165496; doi:10.3389/fmicb.2023.1146496)

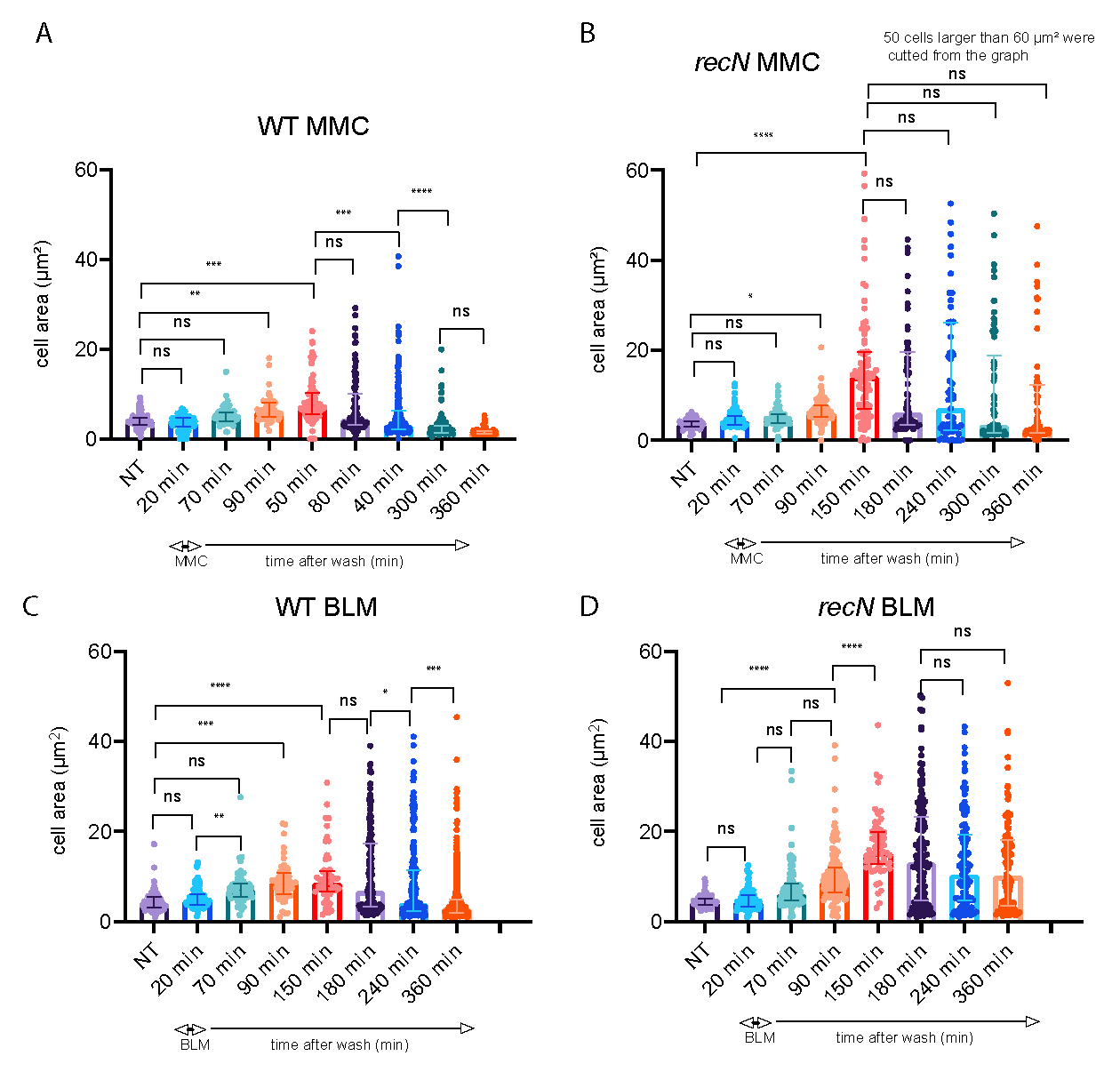

Supplement: Supplementary Figure 1 — Measure of cell area (Phase contrast) during genotoxic treatment (MMC and BLM) and recovery period in WT (A,C) and recN mutant (B,D). N ≈ 400, statistical significance was evaluated by Anova with Türkiye multiple comparison test of the mean cell area (*<0.033, **<0.0021, ***<0.0002, ****<0.0001). [file Image_1.tif]

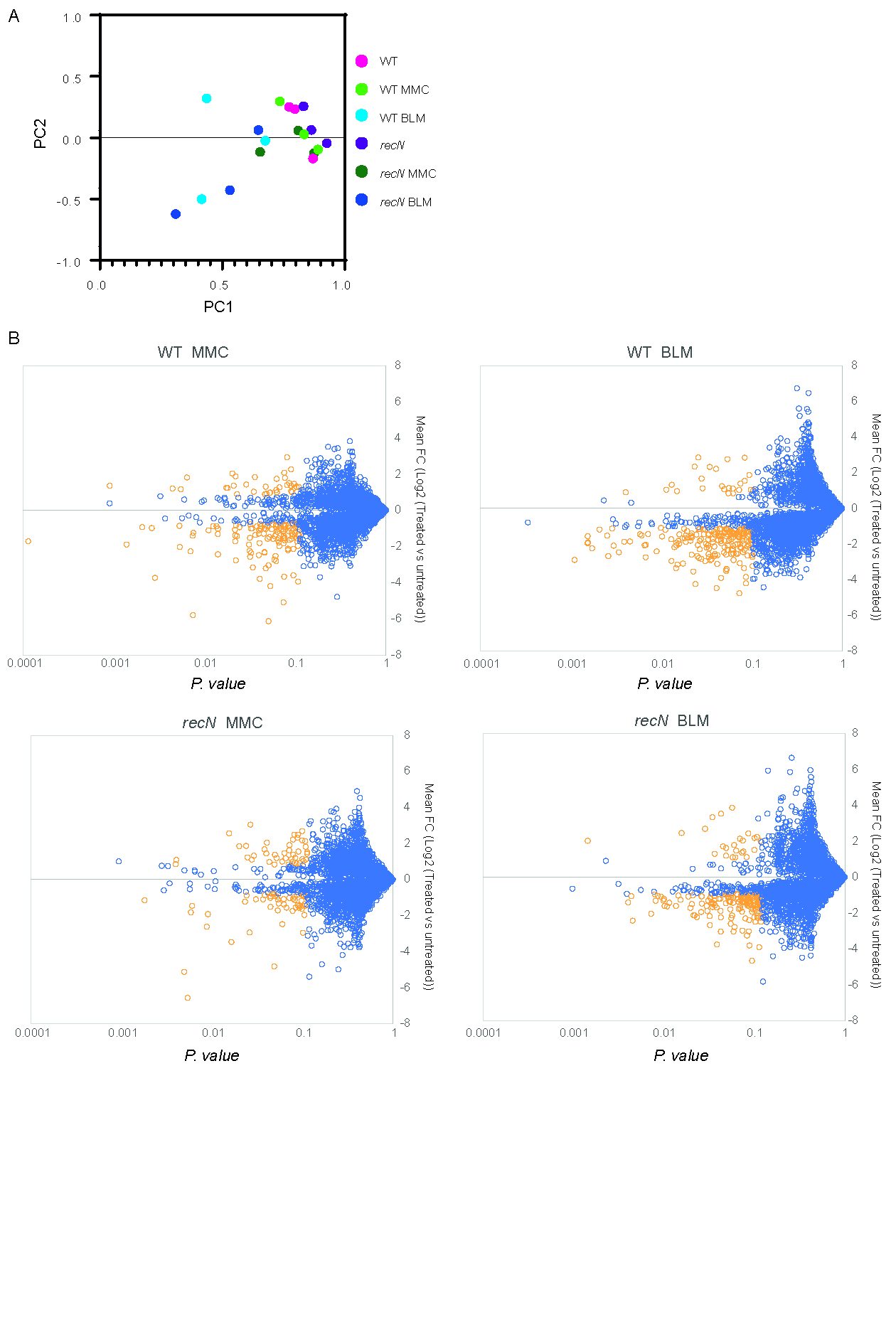

Supplement: Supplementary Figure 2 — (A) Principal component analysis of TIS data. Normalized Insertion Numbers (NIN) were used to compare conditions and replicates. (B) Volcano plot of the mean Log2 FC of TIS replicates and its P-value. Selected genes for the String clustering analysis are colored in orange. [file Image_2.tif]

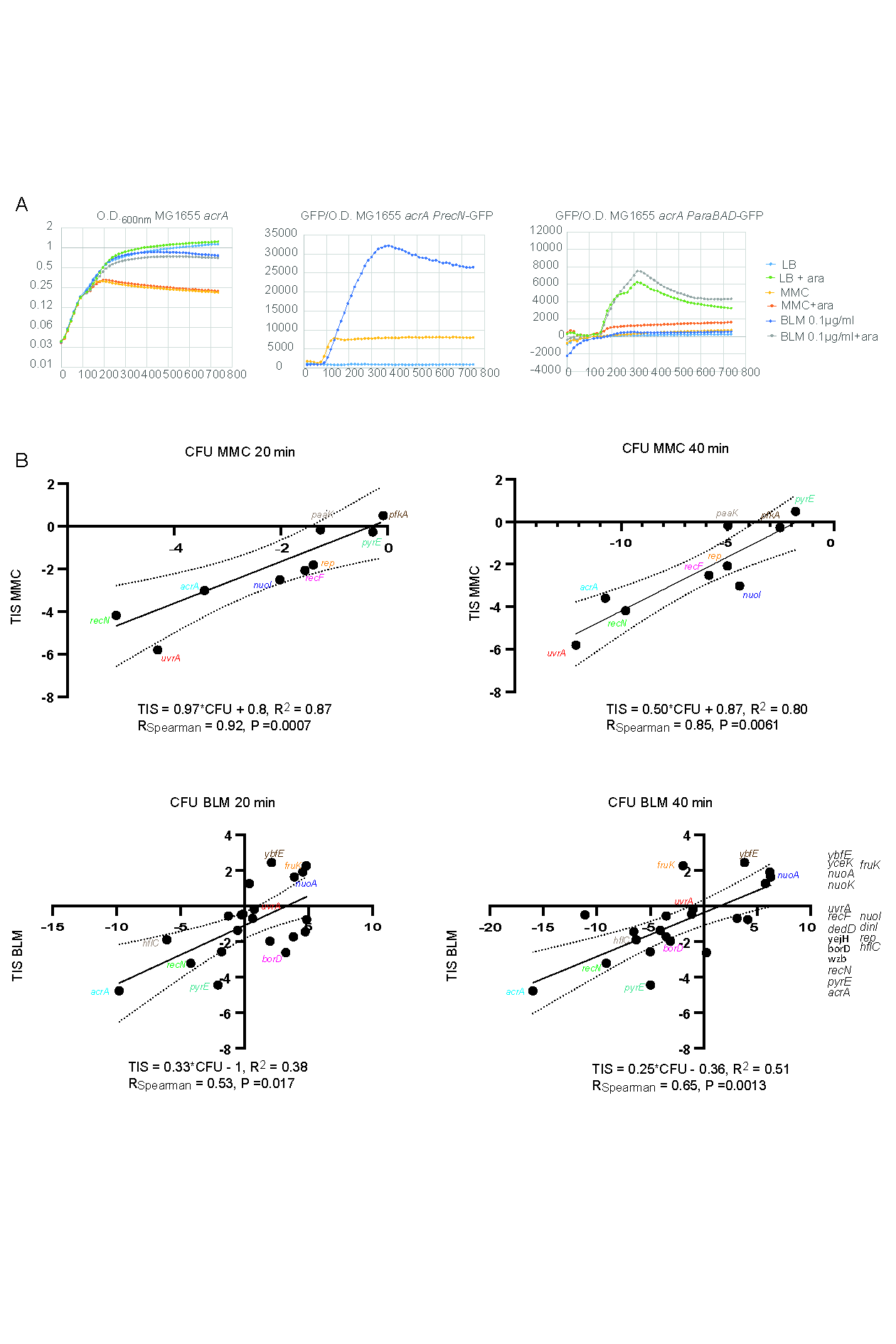

Supplement: Supplementary Figure 3 — (A) Induction of the recN and araBAD promoters in WT and acrA strain in the presence of MMC (5 μg/mL) or BLM (0.1 μg/mL). (B) Comparison of TIS results expressed as mean Log2 FC (NIN treated/NIN untreated) and CFU expressed as mean Log2 FC of the number of colonies in the treated and untreated conditions. Mutants available in the Keio collection were transduced in the WT MG1655 strain and tested after MMC (μg/mL) and BLM (1 μg/mL) treatment. Linear regression (black lines) with 95% confidence intervals (dashed lines) are presented. Pearson (R2) and Sperman coefficient were measured to evaluate de correlation between TIS and CFU data. [file Image_3.tif]

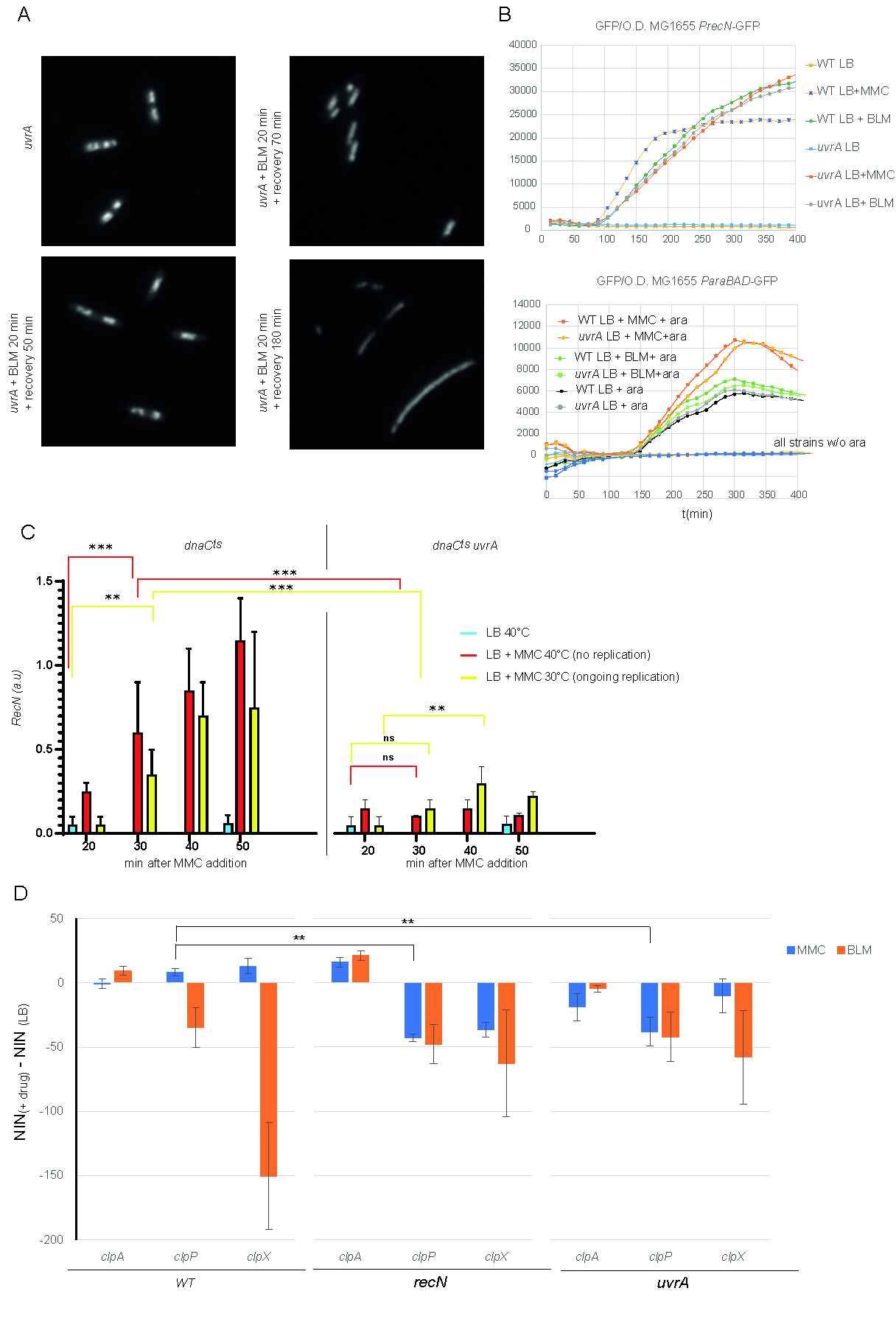

Supplement: Supplementary Figure 4 — (A) Imaging of the nucleoid of the uvrA mutant treated with BLM for 20 min, washed and allowed for recovery. (B) Induction of the recN and araBAD promoter in WT and uvrA strain in the presence of MMC or BLM. (C) RecN production measured by western blot in replicating and non-replicating WT and uvrA mutant cells. To generate non-replicating cells, initiation of replication of dnaCts cells was blocked for 90 min at 40°C before addition of MMC. (D) TIS results for the clpA, clpP, and clpX genes in the WT, recN and uvrA mutants. Data are expressed as the difference between the mean NIN in the treated condition and the mean NIN in the untreated conditions. Multiple paired t-test *<0.1; **<0.05. [file Image_4.tif]
